# Metabolic engineering of *Clostridium cellulolyticum* for the production of *n*-butanol from crystalline cellulose

**DOI:** 10.1186/s12934-015-0406-2

**Published:** 2016-01-13

**Authors:** Stefan Marcus Gaida, Andrea Liedtke, Andreas Heinz Wilhelm Jentges, Benedikt Engels, Stefan Jennewein

**Affiliations:** Department of Industrial Biotechnology, Fraunhofer Institute for Molecular Biology and Applied Ecology, Forckenbeckstrasse 6, 52074 Aachen, Germany

**Keywords:** Metabolic engineering, *Clostridium cellulolyticum*, Biofuels, Butanol, Clostridia

## Abstract

**Background:**

Sustainable alternatives for the production of fuels and chemicals are needed to reduce our dependency on fossil resources and to avoid the negative impact of their excessive use on the global climate. Lignocellulosic feedstock from agricultural residues, energy crops and municipal solid waste provides an abundant and carbon-neutral alternative, but it is recalcitrant towards microbial degradation and must therefore undergo extensive pretreatment to release the monomeric sugar units used by biofuel-producing microbes. These pretreatment steps can be reduced by using microbes such as *Clostridium cellulolyticum* that naturally digest lignocellulose, but this limits the range of biofuels that can be produced. We therefore developed a metabolic engineering approach in *C. cellulolyticum* to expand its natural product spectrum and to fine tune the engineered metabolic pathways.

**Results:**

Here we report the metabolic engineering of *C. cellulolyticum* to produce *n*-butanol, a next-generation biofuel and important chemical feedstock, directly from crystalline cellulose. We introduced the CoA-dependent pathway for *n*-butanol synthesis from *C. acetobutylicum* and measured the expression of functional enzymes (using targeted proteomics) and the abundance of metabolic intermediates (by LC-MS/MS) to identify potential bottlenecks in the *n*-butanol biosynthesis pathway. We achieved yields of 40 and 120 mg/L *n*-butanol from cellobiose and crystalline cellulose, respectively, after cultivating the bacteria for 6 and 20 days.

**Conclusion:**

The analysis of enzyme activities and key intracellular metabolites provides a robust framework to determine the metabolic flux through heterologous pathways in *C. cellulolyticum*, allowing further improvements by fine tuning individual steps to improve the yields of *n*-butanol.

**Electronic supplementary material:**

The online version of this article (doi:10.1186/s12934-015-0406-2) contains supplementary material, which is available to authorized users.

## Background

Liquid transportation fuels are unsurpassed in terms of energy density and they are essential for the transport, haulage and aviation industries. Most liquid transportation fuels and many industrial chemicals are still produced from oil, a fossil resource that is becoming increasingly expensive due to the rising costs of exploration and refinery, and the geopolitical instability in some oil-producing regions. Alternative carbon-neutral energy sources are therefore required to maintain the basis of our technological society without further damage to the environment. Resources such as solar, wind, biomass and geothermal energy production are suitable for static consumers such as homes and workplaces but their conversion to liquid fuels is expensive [1]. Because liquid fuels and green chemicals are necessary for transport and as feedstock for the chemicals industry, there is a tremendous need for the conversion of biomass into green chemicals.

Current biofuel production relies heavily on crops such as maize, but these are also used as food and feed resulting in competition for land and resources [2, 3]. Next-generation biofuels and green chemicals will be produced from lignocellulosic materials, such as agricultural residues, woody energy crops and municipal solid waste, which are abundant and inexpensive [3–5]. The main component of lignocellulose is cellulose, a polymer composed of glucose monomers, but the complex structures of lignocellulose and cellulose make them highly resistant to microbial digestion [6, 7]. Such materials must therefore be pretreated, e.g. by exposure to heat and/or chemicals, followed by digestion with cellulases produced by fungi such as *Trichoderma reesei*, before adding the bacteria or yeast that carry out fermentation (reviewed in [7–9]). These pretreatments tend to be energy-demanding and expensive, cancelling out many of the benefits of renewable energy [8, 10–12].

Several species of anaerobic bacteria can break down cellulose and hemicellulose efficiently, including those of the genera *Clostridium*, *Ruminococcus* and *Thermoanaerobacterium* [13–15]. These bacteria secrete multi-enzyme complexes known as cellulosomes, which convert cellulose into cellobiose and cellodextrins [14–17]. The products are then reabsorbed by the bacteria and metabolized further. One well-characterized example is the model organism is *Clostridium cellulolyticum*, a bacterium that can grow on crystalline cellulose [18–20] and which produces more than 90 glycoside hydrolases (GHs) from different families [21]. The metabolic engineering of cellulose-degrading bacteria to produce specific products would reduce the need for pretreatment, which is the most costly process step [10], and could also reduce the number of steps required to produce biofuels and chemical precursors in a consolidated bioprocess [15, 22, 23].

Butanol is an advanced biofuel with a higher energy content but lower volatility than ethanol, allowing it to be blended with alkanes without the hygroscopicity associated with ethanol blending [5, 24, 25]. Butanol, especially the *n*-butanol isomer, is also an important feedstock for the synthesis of acrylate and methacrylate esters, glycol ethers, butyl acetate, butylamines and amino resins, and it is widely used as a solvent in the chemical industry [5, 24, 26]. Butanol can be produced by anaerobic fermentation using various *Clostridium* species including *C. acetobutylicum* and *C. beijerincki* in acetone–butanol–ethanol (ABE) fermentation processes using monomeric sugars (e.g. molasses) as a feed stock [27–29]. Butanol-producing bacteria such as *C. acetobutylicum* use the coenzyme A (CoA)-dependent pathway to generate *n*-butanol, in which two molecules of acetyl-CoA are condensed to form acetoacetyl-CoA, which is reduced over several further steps to butyryl-CoA, and finally converted to *n*-butanol by a bifunctional alcohol dehydrogenase. This pathway has been introduced by metabolic engineering into several heterologous bacteria, such as *Escherichia coli*, but the resulting *n*-butanol titers were much lower than in *C. acetobutylicum*.

Alternative non-natural pathways involving 2-ketoacid intermediates have also been introduced into *E. coli*, to produce either isobutanol (valine pathway) [30] or *n*-butanol (threonine pathway) [30]. Other organisms have been engineered with these pathways, allowing the production of *n*-butanol or isobutanol from diverse substrates including glycerol, CO/CO_2_, syngas and cellulose (reviewed in [31, 32]). Interestingly, *C. cellulolyticum* can naturally produce isobutanol, but the introduction of the non-native 2-ketoacid pathway increased the overall titers to ~600 mg/L [33]. The 2-ketoacid pathway requires NADPH as a cofactor, but cells generally produce NADH during glycolysis and must therefore produce NADPH either by the direct conversion of NADH using NADH kinase and ATP [34] or via an alternative route such as the pentose phosphate pathway (PPP). Converting hexoses through the PPP to pyruvate results in the net loss of carbon in the form of CO_2_, thus limiting the theoretical yield of butanol.

Here we report for the first time the metabolic engineering of *C. cellulolyticum* with the CoA-dependent pathway to produce *n*-butanol directly from crystalline cellulose. This pathway predominantly uses NADH as a cofactor. The production of *n*-butanol in this manner should not suffer carbon loss like the 2-ketoacid/PPP strategy, so the theoretical yield of butanol from hexoses should be higher when the bacterium is supplied with lignocellulosic material as a source of hexose monomers. The production of* n*-butanol is currently more expensive than ethanol, hence the latter is used more widely, but the ability to produce n–butanol from inexpensive lignocellulosic feedstock means that the process could become more economically feasible [25, 35].

## Results

### Assembly of the CoA-depended pathway for *n*-butanol production

**Fig. 1:**
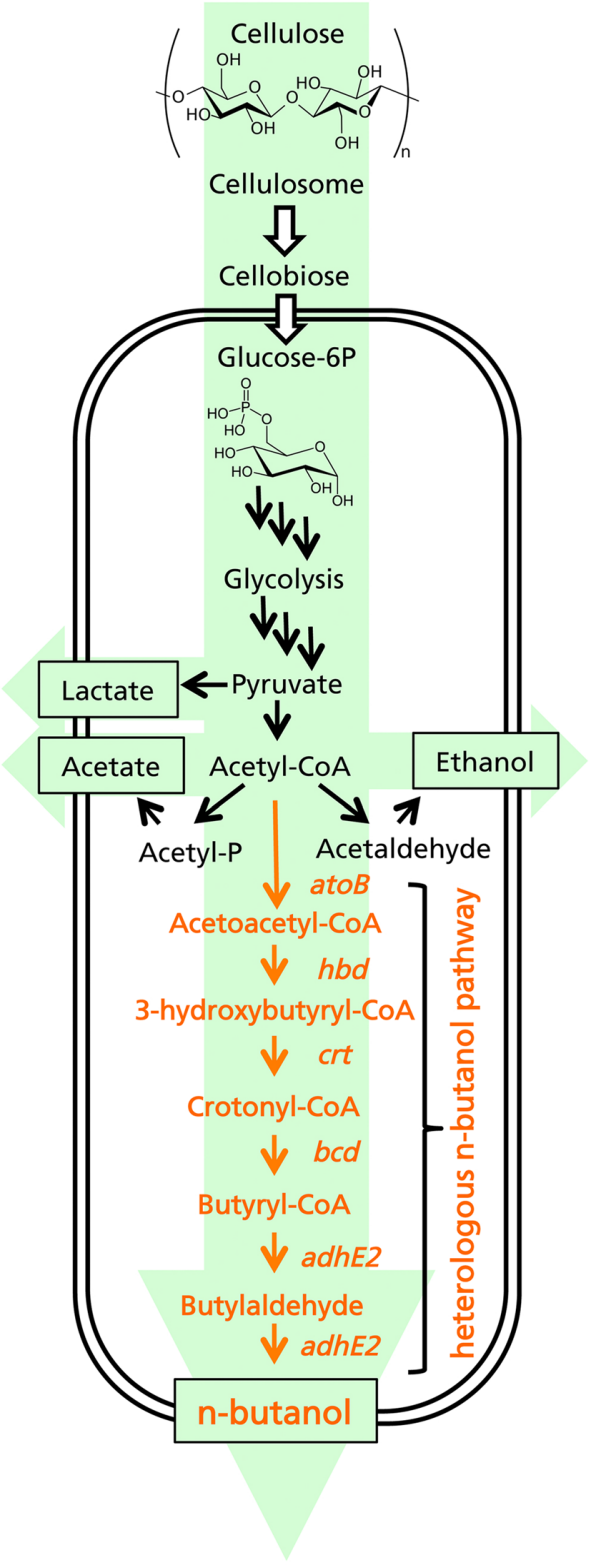
The engineered *n*-butanol pathway, comrising thiolase (*atoB* from *E. coli*), 3-hydroxybutyryl-CoA dehydrogenase (*hbd*), crotonase (*crt*), butyryl-CoA dehydrogenase (*bcd*) and the bifunctional butyraldehyde/butanol dehydrogenase (*adhE2*) (all from *C. acetobutylicum*). Undesirable byproduct reactions include the formation of ethanol (via acetaldehyde and alcoholdehydrogenase), acetate (via phosphotransacetylase and acetate kinase) and lactate (via lactate dehydrogenase)

**Fig. 2:**
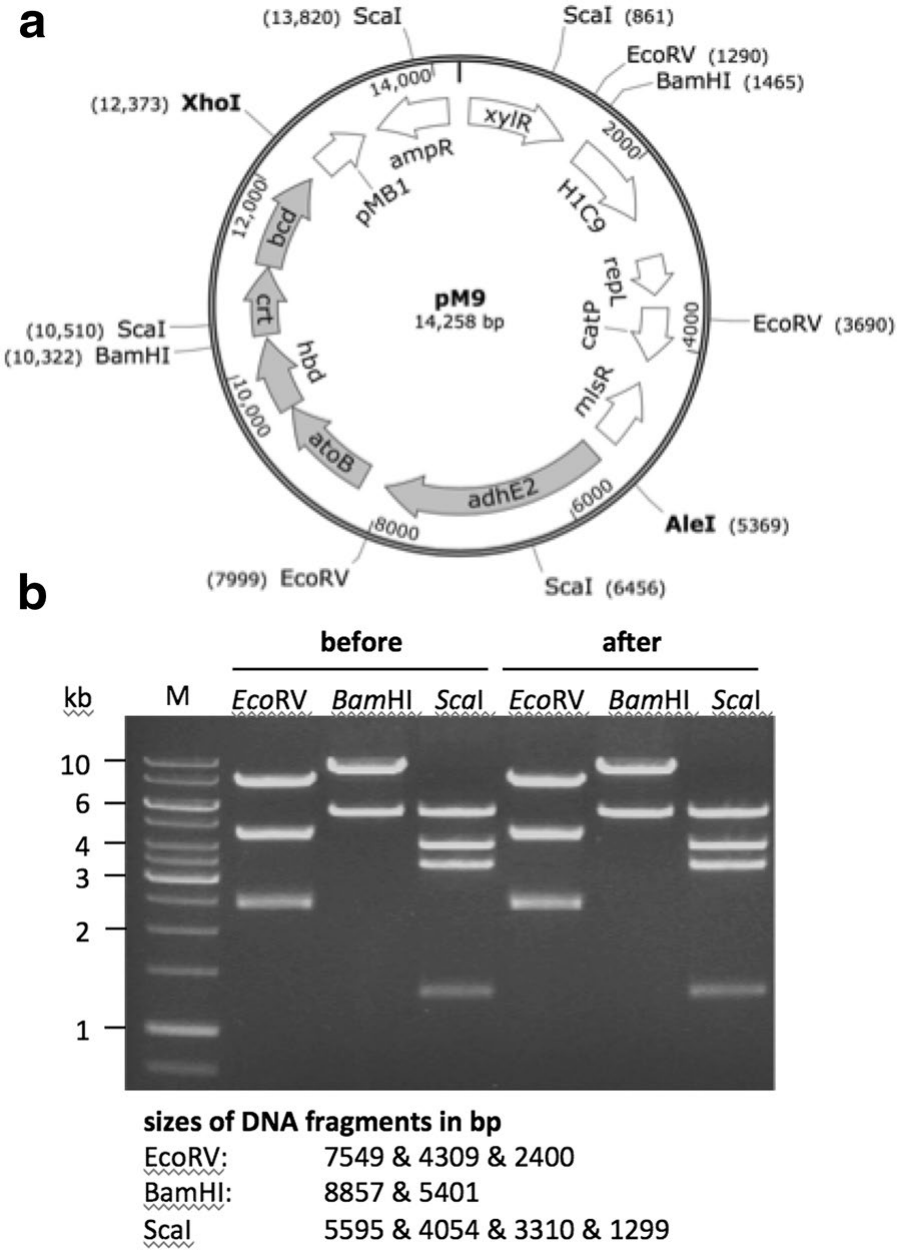
Verification of the pM9 vector. **a** A vector map of pM9 including sites for restriction digest confirmation (*Eco*RV, *Bam*HI and *Sca*I) as well as the insertion sites (*Ale*I and *Xho*I) for the butanol cluster following assembly by SLIC. **b** Confirmation digest of pM9, before and after the transformation of *C. cellulolyticum*

The *E. coliatoB* and *C. acetobutylicumhbd*, *crt*, *bcd* and *adhE2* genes necessary for* n*-butanol production (Fig. 1) were cloned individually in the common *E. coli* expression vector pET-41(a)+ to establish a targeted proteomics strategy allowing the expression of each protein to be confirmed following the transformation of *C.cellulolyticum*. The *E. coliatoB* gene was chosen instead of *C.acetobutylicumthl* because it has a strong track record for the enhancement of butanol production [30] and it is not inhibited by free CoA [36, 37]. The genes were also assembled as a two-operon cluster, comprising a monocistronic operon containing *adhE2* followed by a polycistronic operon containing *atoB*–*hbd*–*crt*–*bcd*, each under the control of the *C. acetobutylicum* thiolase promoter P_thl_. The two-operon strategy was developed to boost the expression of the final gene in the *n*-butanol pathway (*adhE2*) thus creating a metabolic sink and driving the entire pathway towards completion. The clusters were assembled by site and ligation independent cloning (SLIC) and the final cluster was inserted into our in-house *E.coli*/*Clostridium* shuttle vector pIM to create the transformation vector pM9 (Fig. 2). The integrity of pM9 was verified by diagnostic restriction digestion and sequencing (Fig. 2). The pM9 vector was methylated with the MspI methyltransferase from *Moraxella* sp. ATCC 49670 (NEB) and then introduced into *C. cellulolyticum* by electroporation, with successful transformation confirmed by plasmid rescue. This was done to ensure that the complete plasmid was present. We noticed that large plasmids (>10 kb) do not routinely transform *C. cellulolyticum* although shorter derivatives are sometimes present after transformation. Because it is not possible to extract enough plasmid DNA directly from *C. cellulolyticum* to visualize in a restriction digest, the extracted DNA was first introduced back into *E. coli* and isolated from mini-cultures for the restriction digest. This plasmid rescue method is routinely necessary to check for the presence of the intact plasmid as shown in Fig. 2. Verified *C. cellulolyticum* pM9 transformants were then characterized in terms of gene expression, metabolic profiles, growth and product formation on cellobiose and crystalline cellulose substrates as carbon sources.

### Analysis of *n*-butanol cluster proteins in *C. cellulolyticum* strain pM9 using targeted proteomics

The expression of the genes present in the *n*-butanol cluster was confirmed by targeted proteomics. Proteotypic peptides representing each protein (Additional file 1: Table S1) were identified *in silico* using Skyline [38]. Extracts from *E. coli* strains expressing the individual proteins were compared to the proteotypic peptide library to identify the most abundant peptides with the highest signal-to-noise ratios. Extracts from wild-type *E. coli* and *C. cellulolyticum* were used as controls to ensure that the proteotypic peptides were not found in the endogenous proteome. The MS/MS spectrum corresponding to the proteotypic peptides for each gene in the *n*-butanol cluster confirmed that all the peptides could be detected simultaneously and that all five genes were expressed successfully in *C. cellulolyticum* strain pM9 (Additional file 2: Figure S1).

**Fig. 3:**
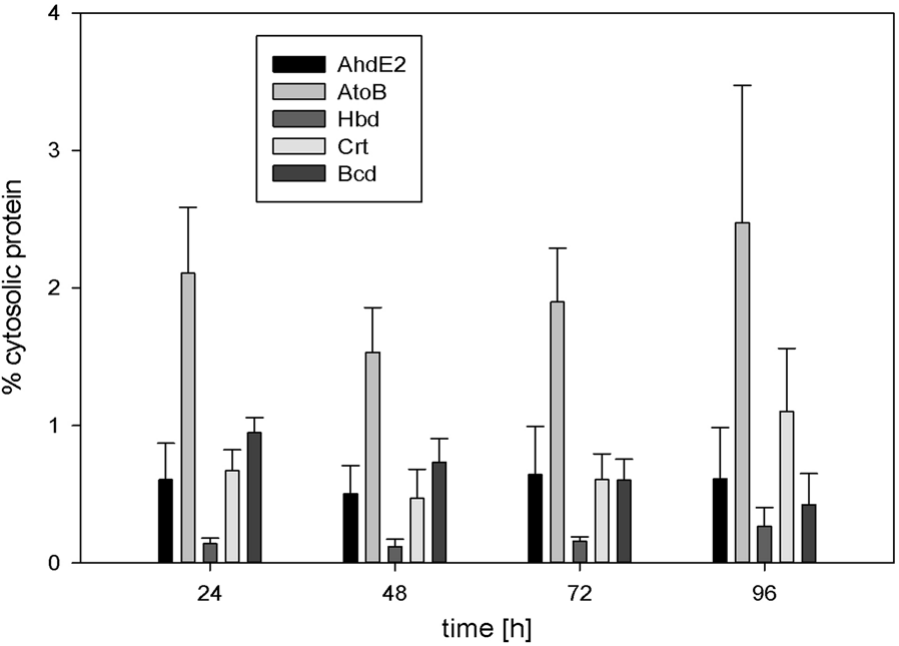
Abundance of the enzymes representing each gene in the *n*-butanol cluster (AtoB, Hbd, Crt, Bcd and AdhE2) during a 96-h fermentation of *C. cellulolyticum* carrying the vector pM9, using cellobiose as the carbon source. *Error bars* represent standard errors of five biological replicates. ANOVA revealed no significant changes (*p* > 0.01) in protein abundance during fermentation, with the exception of a small but significant (*p* < 0.01) decrease for Bcd

Next, we investigated the time-dependent activity of P_thl_ by taking samples throughout the cultivation of *C. cellulolyticum* pM9 up to 96 h post-inoculation, and measuring the abundance of each protein (Fig. 3). All the *n*-butanol pathway proteins were detected during all growth phases, and analysis of variance (ANOVA) revealed no significant change (p > 0.01) in the abundance of most proteins throughout the cultivation. The exception was Bcd, where we noted a small but significant (p < 0.01) decrease in abundance towards the end of the cultivation. The concentration of almost all the proteins therefore remained constant as biomass accumulated during growth, indicating that P_thl_ is constitutively active in *C. cellulolyticum* pM9 and that the proteins are expressed throughout the fermentation process. AtoB was the most abundant protein, followed by Crt, Bcd, AdhE2 and Hbd. It was therefore clear that the construction of a monocistronic operon did not favor the expression of AdhE2 as expected. Because both clusters were controlled by P_thl_, the difference in protein expression was most likely associated with differences in translational efficiency as discussed below.

### Quantification of *n*-butanol pathway intermediates

**Fig. 4:**
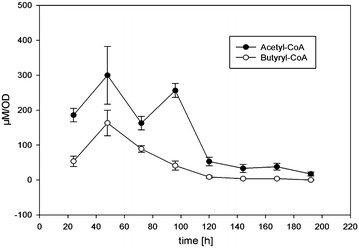
Intracellular pools of **a** acetyl-CoA and **b** butyryl-CoA during a 200-h fermentation of *C. cellulolyticum* carrying the *n*-butanol vector pM9, using cellobiose as the carbon source. The remaining intermediates acetoacetyl-CoA, 3-hydroxybutyryl-CoA and crotonoyl-CoA were not detected. *Error bars* represent standard deviations of three biological replicates

It is important to measure the abundance of pathway intermediates to identify rate-limiting steps, particularly when introducing a novel metabolic pathway into a heterologous host. We therefore developed an analytical method to measure all the intermediates in the* n*-butanol pathway simultaneously, i.e. acetyl-CoA, acetoacetyl-CoA, 3-hydroxybutyryl-CoA, crotonoyl-CoA and butyryl-CoA. We used this method to analyze *C. cellulolyticum* pM9 cultures during a 200-h fermentation. The profiles of acetyl-CoA and butyryl-CoA are shown in Fig. 4, whereas the other intermediates (acetoacetyl-CoA, 3-hydroxybutyryl-CoA and crotonoyl-CoA) were below the detection limit suggesting they were efficiently converted into downstream products. The possibility that the missing intermediates degraded during quenching and extraction was excluded by analyzing samples prepared in an identical manner from wild-type *C. kluyveri*, where all the intermediate CoA esters were detected (data not shown).

### Growth and product formation on cellobiose and crystalline cellulose substrates

The growth of wild-type *C. cellulolyticum* and pM9 transformants was initially measured in CM3 medium containing cellobiose as the carbon source. Cellobiose is a disaccharide comprising two glucose residues linked by a β(1,4) glycosidic bond. Cellobiose is an ideal carbon source to investigate cellulose degradation because the same chemical bond is found in cellulose, but unlike crystalline cellulose it is soluble in water allowing cell growth to be measured by monitoring the turbidity of the suspension. The growth of the two *C. cellulolyticum* strains is compared in Additional file 3: Figure S2. The growth rates were similar in both strains, with doubling times of ~21 h for the wild-type strain and ~20 h for strain containing the pM9 vector. The expression of the *n*-butanol cluster therefore does not appear to generate a metabolic burden that delays growth. Interestingly, doubling times of ~7 h were reported in a variant CM3 medium containing cellobiose, with a fourfold higher concentration of MgCl_2_ and double the concentration of CaCl_2_ [39]. This recipe promotes the formation of precipitates, which we avoided by using lower concentrations of these minerals, but nevertheless the report demonstrates that medium optimization can improve growth and therefore establish a faster bioprocess.

**Fig. 5:**
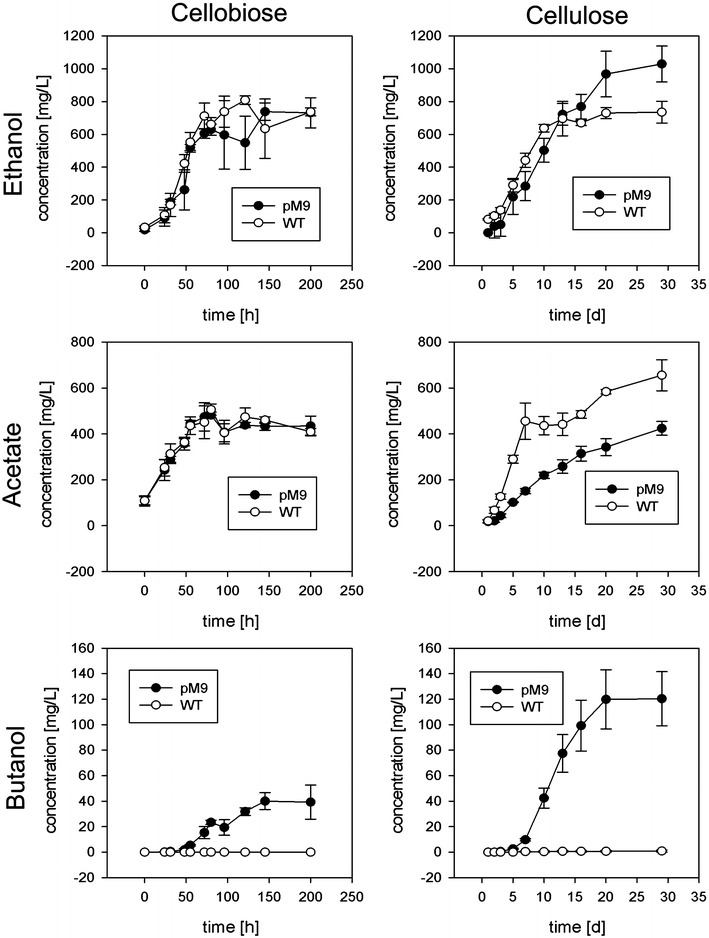
Product formation by *C. cellulolyticum* wild-type (WT) and strain pM9 during cultivation using cellobiose (10-day fermentation) or crystalline cellulose (30-day fermentation) as the sole carbon source. *Error bars* represent standard deviations of three independent biological replicates

Next, we compared product formation by the two strains when supplied with cellobiose or crystalline cellulose. As expected, the wild-type *C. cellulolyticum* cultures produced ethanol and acetate as the main fermentation products rather than *n*-butanol. In contrast, strain pM9 produced *n*-butanol in addition to ethanol and acetate (Fig. 5). Importantly, strain pM9 produced more *n*-butanol when cultivated in the presence of crystalline cellulose rather than cellobiose as the sole carbon source (Fig. 5). This, to our knowledge, is the first time that* n*-butanol has been produced directly from crystalline cellulose using a single organism. We achieved titers of 40 and 120 mg/L *n*-butanol in cultures supplied with cellobiose and crystalline cellulose, respectively.

### Sugar consumption

**Fig. 6:**
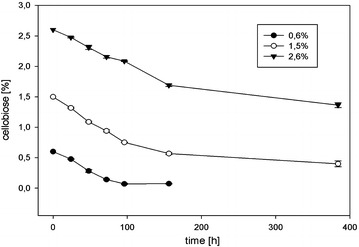
Cellobiose consumption by *C. cellulolyticum* pM9 strain during cultivation using different initial cellobiose concentrations ranging from 0.6 to 2.6 % (w/v). *Error bars* represent standard deviations of three biological replicates

We attempted to increase the productivity of *C. cellulolyticum* pM9 cultures growing on cellobiose by increasing the concentration of the substrate, but there were no significant differences in the consumption of cellobiose in stationary-flask fermentations starting with cellobiose concentrations of 6, 15 and 26 g/L (Fig. 6). Regardless of the initial amount of cellobiose, only ~6 g/L of the substrate was consumed. This suggests that the ability to utilize cellobiose as a substrate is a rate-limiting step in the production of *n*-butanol.

## Discussion

The construction of entire heterologous pathways for metabolic engineering in non-model organisms, such as *C. cellulolyticum* described herein, can be challenging because the genetic toolbox available for this Gram-positive species is not as sophisticated as those available for laboratory models such as *E. coli* despite recent advances in this area (reviewed in [29, 40]). Nevertheless, we were able to construct and express a functional *n*-butanol pathway in *C. cellulolyticum* as confirmed by a targeted proteomics strategy designed to detect all five heterologous proteins, as well as the identification of some of the pathway intermediates and products. The targeted proteomics method will also be useful for the optimization of the engineered strain because it will allow the impact of genetic modifications (e.g. different promoters or ribosomal binding sites) to be monitored at the protein level. Such modifications are typically monitored by northern blotting, RNase protection assays, microarray analysis or quantitative RT-PCR, but these methods only reveal changes at the mRNA level. The direct quantitative analysis of proteins (e.g. by western blotting or ELISA) requires specific antibodies and is laborious and expensive to apply in a multiplex format. Our targeted proteomics strategy allows the simultaneous direct quantitation of multiple enzymes and therefore allows the impact of genetic changes on translational efficiency and protein turnover to be reported directly.

The *n*-butanol cluster was constructed as two operons driven by the P_thl_ promoter, specifically an upstream monocistronic operon containing *adhE2* and a downstream polycistronic operon containing the other four genes, starting with *atoB*. We therefore anticipated that *adhE2* and *atoB* would be expressed at similar levels, but this was not the case. One possible explanation is that the resulting mRNAs are translated with different efficiencies because they contain distinct ribosomal binding sites (AGGAGG for *atoB* and AGGAGU for *adhE2*). As discussed above, the targeted proteomics strategy could be used to evaluate modifications of the ribosomal binding site, 5′ untranslated region or codon usage, all of which are known to influence translational efficiency and mRNA stability [41–44] therefore greatly facilitating the further optimization of engineered *C. cellulolyticum* strains.

The commercial production of *n*-butanol using traditional ABE fermentation is carried out in China by Jilin Cathy Industries at a total cost of ~$US 2000 per ton, with most of the costs (~70 %) reflecting the use of maize as the feedstock [35]. The optimization of the current pM9 strain is necessary because the final product titers are low. We achieved a final titer of 120 mg/L *n*-butanol from 12 g/L crystalline cellulose (Fig. 5) which is clearly not sufficient for the industrial production of fuels or chemicals, but it is equivalent to or better than *n*-butanol titers reported when feeding engineered strains with other lignocellulosic or renewable substrates. For example, yields of up to 28 mg/L have been achieved using switch grass [45], 25–300 mg/L using glycerol [46, 47], up to 148 mg/L using syngas [48] and ~400 mg/L using CO_2_ and light [49]. Interestingly, *C. cellulolyticum* can naturally produce isobutanol under certain growth conditions, and this can be boosted to titers of 660 mg/L by incorporating the non-natural 2-ketoacid pathway [33]. The engineered 2-ketoacid pathway uses NADPH, an anabolic cofactor that is not formed during glycolysis, whereas the CoA-dependent pathway uses NADH [33]. Cells using the 2-ketoacid pathway must therefore convert NADH to NADPH or use the PPP as an alternative route for the production of isobutanol. In the PPP, carbon is lost due to decarboxylation, therefore limiting the theoretical yield of isobutanol. *Clostridium* spp. express a ferredoxin-NADP^+^ oxidoreductase that converts NADP^+^ to NADPH+H^+^ by oxidizing ferredoxin [50]. Nevertheless, NADH^+^H^+^ is the primary electron accepter during glycolysis and the cell therefore needs an effective way to transfer electrons from NADH^+^H^+^ to ferredoxin and ultimately back to NADP^+^. This could be accomplished directly using the electron-bifurcation transhydrogenase (Nfn complex) initially identified in *C. kluyveri* and more recently also found in acetogenes such as *C. ljungdahlii*, *C. autoethanogenum* and *Acetobacterium woodii* [51], but not thus far in *C. cellulolyticum*.

As a starting point, we therefore introduced the Co-A dependent pathway into *C. cellulolyticum*. With further improvement of the pathway (based on information generated using our robust framework of targeted proteomics and the detection of intermediates) as well as host strain engineering (e.g. the knockout of genes producing undesirable byproducts such as lactate) it should be possible to increase* n*-butanol production even more. The CoA-dependent and 2-ketoacid pathways could also be combined, increasing the overall production of both butanol isomers when the engineered bacteria are presented with lignocellulosic feedstocks, because C5 sugars in the lignocellulosic material could enter the PPP directly (without carbon loss) and generate the NADPH required for the 2-ketoacid pathway.

Nevertheless, the yields we achieved are much lower than the those reported using traditional sugar-based feedstocks such as glucose, galactose and mannitol, where titers of up to 30 g/L have been reported following strain and process optimization [52]. Host cell engineering is necessary to redirect flux towards *n*-butanol and reduce the production of undesirable byproducts [30, 45, 53, 54]. Targets reported in the literature, which also apply to *C. cellulolyticum*, include genes involved in the production of ethanol (Δ*adhE*), acetate (Δ*pta* and Δ*ack*) and lactate (Δ*ldh*). Thus far, it has been possible to eliminate the production of lactate but not in *C. cellulolyticum* [55].

## Conclusion

To our knowledge, this is the first report in which *n*-butanol has been produced directly from crystalline cellulose using a single engineered organism. This is an important finding because it allows the development of an inexpensive, consolidated bioprocess with fewer and milder pretreatment steps [23]. In addition to the expense, energy consumption and environmental harm caused by pretreatment [7, 10, 55–57], such processes also tend to release inhibitors such as furan derivatives, weak carboxylic acids and phenolic compounds that interfere with downstream fermentation [7, 58]. Thus with milder pretreatment conditions the quantity of such inhibitors could be reduced and fermentation performance could be improved. Strategies to facilitate the development of consolidated bioprocess include the genetic engineering of natural cellulolytic microbes to produce desired products (as reported here) or to enable the fermentation of lignocellulosic feedstock with established production organisms [23]. Furthermore, processes have been developed which involve synergistic combinations of bacterial strains with different roles in the breakdown and utilization of cellulose [45, 59]. This approach is challenging because the different bacteria compete with each other and it is difficult to keep the different populations in balance, whereas the use of a single engineered *C. cellulolyticum* strain improves the potential yield by allowing the process to be optimized for one bacterial population. However, the characteristics of the process (including the product titer and avoidance of byproducts) must be optimized to ensure the efficient conversion of biomass into fuels and chemicals. The further improvement of our strain to meet these criteria will be facilitated by our combined approach based on targeted proteomics and metabolite analysis.

## Methods

### Bacterial strains and plasmids

The *E. coli* strain NEB Express (NEB, Ipswich, MA, USA) was used for general cloning with vector pET-41a(+) (Novagen, Darmstadt, Germany). We developed a shuttle vector (pIM) for the transfer of constructs between *E. coli* stains and the *C. cellulolyticum* wild-type strain H10 (DSZM, Braunschweig, Germany) as well as vector pM9 containing the *n*-butanol gene clusters.

### Media composition and cultivation

The *E. coli* strains were cultivated in lysogeny broth at 37 °C shaking at 220 rpm. The *C.cellulolyticum* stains were cultivated in CM3 medium, comprising 1.3 g/L (NH_4_)_2_SO_4_, 1.5 g/L KH_2_PO_4_, 2.9 g/L K_2_HPO_4_∙3H_2_O, 0.2 g/L MgCl_2_∙6H_2_O, 0.075 g/L CaCl_2_∙2H_2_O, 1.25 mg/L FeSO_4_∙7H_2_O, 1 mg/L resazurin, 2 g/L yeast extract, 0.5 g/L cysteine and either 6 g/L cellobiose or 12 g/L crystalline cellulose (Avicel PH101) as the carbon source. The pH was adjusted to 7.2 with 5 % Na_2_CO_3_ after sterilization and the medium was deoxygenated under an anaerobic atmosphere (5 % H_2_, 10 % CO_2_, 85 % N_2_). *C. cellulolyticum* was grown under anaerobic conditions at 34 °C. For the preparation of agar plates, the media described above were supplemented with 15 g/L agar.

### Cloning the butanol cluster

The genes for the butanol cluster were sourced from two bacterial species: *hbd*, *crt*, *bcd* and *adhE2* from *C. acetobutylicum* and *atoB* from *E. coli*. They were amplified from genomic DNA by PCR and transferred to the pET-41a(+) vector by SLIC [60]. The same procedure was used to assemble the genes into clusters, one comprising the *C. acetobutylicum* thiolase promoter (P_thl_) followed by *atoB*, *hbd*, *crt* and *bcd*, and the other comprising P_thl_ followed by *adhE2*. The clusters were then combined to place the *adhE2* gene upstream of the others, and the entire construct was introduced into our in-house Gram^+/−^ shuttle vector pIM at the *Xho*I and *Ale*I sites. The final vector pM9 was sequenced to ensure correct assembly. A map of the vector is shown in Fig. 2.

### Transformation of *C. cellulolyticum*

DNA was introduced into *C. cellulolyticum* by electroporation [61]. Cells in the late exponential growth phase (10–50 mL, OD_600_ = 0.5–1) were chilled on ice for 30 min before centrifuging (4000×*g* for 10 min) and washing twice with ice-cold electroporation buffer (5 mM sodium phosphate buffer pH 7.4, 270 mM sucrose, 1 mM MgCl_2_) before resuspending in 200 µl of the same buffer. The competent cells were mixed with 1–5 µg of in vitro methylated DNA and transferred to a 0.2-mm gap cuvette (BioRad, Hercules, CA, USA) before electroporation with a BioRad Micropulser set at 1.5 kV. DNA was methylated with MspI methyltransferase (NEB) for at least 4 h according to the manufacturer’s instructions. The cells were allowed to recover in CM3 medium without antibiotics for 4–6 h before plating on medium containing 5 µg/L clarithromycin and 10 µg/L thiamphenicol. All procedures except the electroporation step were carried out under anaerobic conditions with anoxic solutions. Successful transformation was verified by plasmid rescue followed by a diagnostic restriction digest.

### Product analysis

Fermentation products were quantified by GC/MS on a Shimadzu GCMS-QP2010S system (Shimadzu, Kyoto, Japan). The culture supernatant was diluted 1:10 in methanol containing 5.5 mM 1,3-propanediol as an internal standard. The temperature profile of the GC protocol included an initial 1-min step at 60 °C followed by a temperature gradient of 5 °C/min to 70 °C and 35 °C/min to 220 °C, and then a 2-min hold before cooling to 60 °C for the next run. Retention times and the quantification of ethanol, butanol and acetate were established using GC/MS-grade standards.

### Sample preparation for targeted proteomics

Cells were harvested by centrifugation at 4000×*g* for 15 min at 4 °C, the supernatant was discarded and the pellet resuspended in 1 mL 50 mM ammonium bicarbonate (pH 7.8). The suspension was transferred to a 2-mL steel reaction tube, mixed with 0.5 g of 0.1-mm glass beads and shaken vigorously for 3 × 30 s on a bead beater (Biospec Products, Bartlesville, OK, USA) with intervening 1-min incubations on ice. The supernatant was clarified by centrifugation (13,000×*g*, 5 min, 4 °C) and the protein concentration was determined using Bradford Quick-Start Reagent (BioRad) against a bovine serum albumin standard curve. We then transferred 200 µg of total protein to ammonium bicarbonate buffer containing 0.05 % PPS silent sur-factant (Expedeon, San Diego, CA, USA), boiled the samples in a water bath for 5 min and reduced the proteins by adding 5 mM dithiothreitol and incubating at 60 °C for 30 min. The sample was alkylated using 15 mM iodoacetic acid at 25 °C for 30 min in the dark. The denatured protein was digested with sequencing-grade modified trypsin (Promega, Mannheim, Germany) at an enzyme:substrate ratio of 1:100 at 37 °C with vigorous shaking for at least 16 h. The peptide mix was desalted using 1-mL (30 mg) Chromabond HR-X cartridges (Macherey Nagel, Dueren, Germany) conditioned with 1 mL acetonitrile and 1 mL double-distilled water. After adding the sample, the cartridge resin was washed twice with 1 mL double-distilled water and the peptides were eluted by the stepwise addition of 250 µL 40:60 acetonitrile/water, 70:30 acetonitrile/water and finally 70:30 acetonitrile/1.0 % formic acid. The elution fractions were pooled and concentrated to a volume of ~50 µL using a SpeedVac (Eppendorf, Hamburg, Germany) at 45 °C. The samples were adjusted to a final volume of 100 µL with 0.1 % formic acid.

### Prediction of peptide mass transitions

For each target protein, peptide mass transitions were predicted using Skyline [38] ignoring the first 25 amino acids and including all peptides 7–14 amino acids in length assuming the carbamidomethylation of cysteine residues. The declustering, entrance potential and collision energy parameters were calculated using Skyline for AB Sciex instruments. The sum of all dwell times for a maximum of 100 transitions in one run did not exceed 1 s/cycle to obtain at least 10 data points per signal. As a positive control for fusion protein expression, we monitored the proteotypic peptides LLLEYLEEK and IEAIPQIDK [62] derived from the glutathione-S-transferase (GST) protein fusion part.

### Mass spectrometry

Peptide analysis was carried out using a 3200 QTRAP triple-quadrupole analyzer (AB Sciex, Framingham, MA, USA) in electrospray ionization mode. Mixtures were separated by high performance liquid chromatography (HPLC) using an Agilent 1200 instrument (Agilent Technologies, Santa Clara, CA, USA) and an EC 150/2 Nucleoshell RP18, 2.7 µm column (Macherey Nagel) with solvent A (5 % acetonitrile/95 % water containing 0.1 % formic acid) and solvent B (95 % acetonitrile/5 % water containing 0.1 % formic acid). After 1 min at 0 % B the gradient was increased to 30 % B in 30 min and then to 100 % B in 5 min before an isocratic run at 100 % B for 8 min and final re-equilibration of the column (total run time, 60 min). To determine the proteotypic peptides, each GST-protein fusion was expressed and analyzed separately. The monoisotopic peptide mass in the +2 state was selected in Q1, and after fragmentation with peptide-specific collision energy in q2, the monoisotopic fragment y-ions in the +1 state were selected in Q3. The specific masses for this multiple reaction monitoring (MRM) of each proteotypic peptide are provided in Additional file 2: Figure S1. Two MRM ions were selected per proteotypic peptide for identification (Qualifier) and quantification (Quantifier).

### Peptides

After the selection of suitable proteotypic peptides (Additional file 2: Figure S1), heavy [^13^C_6_^15^N_2_]-labeled peptides were used as internal standards (SpikeTides L™, JPT Peptide Technologies, Berlin, Germany). Heavy lysine (L) and arginine (R) labeling results in mass shifts of +8 (L) and +10 (R) atomic mass units. The corresponding transitions for internal standards were incorporated into our calculations. The absolute quantification of selected peptides was achieved using Q-Tag fusions (SpikeTides TQ™, JPT Peptide Technologies) according to the manufacturer’s recommendations. The 1 nmol stock of labeled Q-Tag fusion peptides was digested and desalted as described above for the extracted protein samples. Afterwards a dilution series was prepared and analyzed to calculate a standard curve used for absolute quantification. The specific masses for the MRM ions for these heavy (labeled) proteotypic peptides are provided in Additional file 2: Figure S1.

### Intracellular metabolite analysis by LC/MS/MS

Intracellular metabolites were extracted by quenching the biomass using an acidic solvent mixture [63] and lysing the cells by vortexing on ice for 10 min. The lysate was clarified by centrifugation (13,000×*g*, 5 min, 4 °C) and the supernatant was neutralized with an equimolar volume of ammonium hydroxide before separation by HPLC on a Kinetex 2.6 µm C18 column 150 × 4.6 mm (Phenomenex, Aschaffenburg, Germany) as previously described [64]. The metabolites were analyzed using a 3200 QTRAP triple-quadrupole MS (AB Sciex) in electrospray negative ionization mode. Metabolite references (acetyl-CoA, acetoacetyl-CoA, 3-hydroxybutyryl-CoA, crotonyl-CoA, butyryl-CoA and octanoyl-CoA) were tuned by direct infusion and a standard curve for correlation was generated with 3.125 µM as the lowest level showing a good signal-to-noise ratio. After flow injection optimization, quantitative analysis was carried out by MRM with octanoyl-CoA (50 µM) as an internal standard. The following transitions were used: acetyl-CoA, 807.962 → 408.000, 807.962 → 425.900, 807.962 → 461.000; acetoacetyl-CoA, 850.100 → 408.100, 850.100 → 418.900, 850.100 → 765.900; 3-hydroxybutyryl-CoA, 852.100 → 408.000, 852.100 → 426.000, 852.100 → 505.000; crotonyl-CoA, 834.100 → 408.000, 834.100 → 426.000, 834.100 → 487.000 butyryl-CoA, 835.962 → 408.000, 835.962 → 425.9, 835.962 → 489.000; octanoyl-CoA, 891.970 → 408.000, 891.970 → 425.900, 891.970 → 545.200.

### Sugar analysis

Quantitative analysis of the carbon source in the culture supernatant was carried out using a Prominence HPLC System and refractive index detector (Shimadzu). Cellobiose, glucose and xylose were separated on a Rezex RCM Monosaccharide Ca^+2^ (8 %) 300 × 7.8 mm column (Phenomenex) in an isocratic run at 80 °C with double-distilled water as the solvent and a flow rate of 0.6 mL/min. Supernatant samples filtered and diluted with an equal volume of sorbitol (2 % v/v) were used as an internal standard.

## Additional files

10.1186/s12934-015-0406-2 Proteotypic peptides representing each gene in the n-butanol cluster.
10.1186/s12934-015-0406-2 MS/MS spectra of the proteotypic peptides representing each gene in the n-butanol cluster.
10.1186/s12934-015-0406-2 Growth curve of *C. cellulolyticum* wild-type and pM9 strains on cellobiose during a 150-h fermentation, determined by the measurement of optical density at 600 nm (OD_600nm_). Error bars represent standard deviations of three biological replicates.
