# Supplementary material for: Metabolic engineering of Clostridium cellulolyticum for the production of n-butanol from crystalline cellulose
Source: Microb Cell Fact. 2016 Jan 13;15:6. doi: 10.1186/s12934-015-0406-2 (PMC4711022; doi:10.1186/s12934-015-0406-2)
Supplement: Supplementary file 1 — 10.1186/s12934-015-0406-2 Proteotypic peptides representing each gene in the n-butanol cluster. [file 12934_2015_406_MOESM1_ESM.docx]

**Table S1.** Proteotypic peptides representing each gene in the n-butanol cluster.

| **Protein** | **Proteotypic peptide** |
| --- | --- |
| AtoB | LGDGQVYDVILR |
| HbD | GFEVVLR |
| Crt | VAVVTINRPK |
| Bcd | YLVPLAK |
| AdhE2 | VTALIEAISK |
