# Supplementary material for: Metabolic engineering of Clostridium cellulolyticum for the production of n-butanol from crystalline cellulose
Source: Microb Cell Fact. 2016 Jan 13;15:6. doi: 10.1186/s12934-015-0406-2 (PMC4711022; doi:10.1186/s12934-015-0406-2)
Supplement: Supplementary file 2 — 10.1186/s12934-015-0406-2 MS/MS spectra of the proteotypic peptides representing each gene in the n-butanol cluster. [file 12934_2015_406_MOESM2_ESM.docx]

**Figure S1.** MS/MS spectra of the proteotypic peptides representing each gene in the n-butanol cluster.
