# Supplementary material for: Metabolic engineering of Clostridium cellulolyticum for the production of n-butanol from crystalline cellulose
Source: Microb Cell Fact. 2016 Jan 13;15:6. doi: 10.1186/s12934-015-0406-2 (PMC4711022; doi:10.1186/s12934-015-0406-2)
Supplement: Supplementary file 3 — 10.1186/s12934-015-0406-2 Growth curve of C. cellulolyticum wild-type and pM9 strains on cellobiose during a 150-h fermentation, determined by the measurement of optical density at 600 nm (OD600nm). Error bars represent standard deviations of three biological replicates. [file 12934_2015_406_MOESM3_ESM.docx]

**Figure S2.** Growth curve of *C. cellulolyticum* wild-type and pM9 strains on cellobiose during a 150-h fermentation, determined by the measurement of optical density at 600 nm (OD_600nm_). Error bars represent standard deviations of three biological replicates.
